# High glucose suppresses embryonic stem cell differentiation into cardiomyocytes: High glucose inhibits ES cell cardiogenesis

**DOI:** 10.1186/s13287-016-0446-5

**Published:** 2016-12-09

**Authors:** Penghua Yang, Xi Chen, Sunjay Kaushal, E. Albert Reece, Peixin Yang

**Affiliations:** 1 Department of Obstetrics, Gynecology and Reproductive Sciences, University of Maryland School of Medicine, BRB11-039, 655W. Baltimore Street, Baltimore, MD 21201 USA; 2 Division of Cardiac Surgery, University of Maryland School of Medicine, Baltimore, MD 21201 USA; 3 Department of Biochemistry and Molecular Biology, University of Maryland School of Medicine, Baltimore, MD 21201 USA

**Keywords:** Embryonic stem cells, Cardiogenesis, High glucose, Contracting cardiomyocytes

## Abstract

**Background:**

Babies born to mothers with pregestational diabetes have a high risk for congenital heart defects (CHD). Embryonic stem cells (ESCs) are excellent in vitro models for studying the effect of high glucose on cardiac lineage specification because ESCs can be differentiated into cardiomyocytes. ESC maintenance and differentiation are currently performed under high glucose conditions, whose adverse effects have never been clarified.

**Method:**

We investigated the effect of high glucose on cardiomyocyte differentiation from a well-characterized ESC line, E14, derived from mouse blastocysts. E14 cells maintained under high glucose (25 mM) failed to generate any beating cardiomyocytes using the hanging-drop embryonic body method. We created a glucose-responsive E14 cell line (GR-E14) through a graduated low glucose adaptation. The expression of stem cell markers was similar in the parent E14 cells and the GR-E14 cells.

**Results:**

Glucose transporter 2 gene was increased in GR-E14 cells. When GR-E14 cells were differentiated into cardiomyocytes under low (5 mM) or high (25 mM) glucose conditions, high glucose significantly delayed the appearance and reduced the number of TNNT2 (Troponin T Type 2)-positive contracting cardiomyocytes. High glucose suppressed the expression of precardiac mesoderm markers, cardiac transcription factors, mature cardiomyocyte markers, and potassium channel proteins. High glucose impaired the functionality of ESC-derived cardiomyocytes by suppressing the frequencies of Ca^2+^ wave and contraction.

**Conclusions:**

Our findings suggest that high glucose inhibits ESC cardiogenesis by suppressing key developmental genes essential for the cardiac program.

**Electronic supplementary material:**

The online version of this article (doi:10.1186/s13287-016-0446-5) contains supplementary material, which is available to authorized users.

## Background

Nearly 2% of all pregnancies are affected by pregestational diabetes [1]. Infants from pregestational diabetic mothers have a greater risk of structural birth defects including congenital heart defects (CHDs) than those from nondiabetic mothers [2]. CHDs are the most common congenital anomalies occurring in approximately 4 to 10 per 1000 live births [3]. Our previous studies have shown that pregestational diabetes induces oxidative stress and activates pro-apoptotic kinase signaling leading to endoplasmic reticulum stress, impaired cell proliferation, and apoptosis, which cause defective heart formation [4–12]. Additionally, maternal diabetes represses the expression of transcription factors that are essential for cardiac lineage specification [5, 8, 9, 13], suggesting that maternal diabetes adversely impacts cardiogenesis during early embryonic development. The objective of the present study is to study the effect of high glucose on ESC differentiation into cardiomyocytes.

Pluripotent embryonic stem (ES) cells, derived from the preimplantation embryo, can differentiate into derivatives of all three primary germ layers [14]. Since the first mouse and human ES cells were successfully isolated from blastocysts [15, 16], they have been widely used for the generation of differentiated cells including cardiomyocytes [17–20]. The ES differentiation process to cardiomyocytes is a unique in vitro model for studying the effect of high glucose on early cardiac lineage specification and cardiomyocyte maturation. Several procedures have been reported for cardiomyocyte differentiation from ES cells with different efficiencies [21–24]. ES differentiation procedures using kinase inhibitors or inhibitors of the Wnt signaling achieve a high yield of positive cardiomyocytes [25]; however, these inhibitors may adversely impact the function of resultant cardiomyocytes. Using the embryoid body (EB) hanging-drop method, a low number of contracting cardiomyocytes can be generated in regular medium without small molecules and kinase inhibitor treatment. Therefore, we used this method in our experiment.

Essentially, all ES cell differentiation procedures have been performed under high glucose conditions (25 mM), which is not comparable to physiological concentration of glucose (5 mM) [26]. ES cell differentiation into cardiomyocytes under physiological glucose conditions recapitulates the cardiac cell lineage specification during early embryogenesis. In the present study, the mouse ES cell line (E14), initially cultured under high glucose, was gradually adapted into low glucose conditions and eventually maintained under 5 mM glucose conditions. The stemness of these adapted E14 cells, which were glucose responsive (GR-E14 cells), was not different with that of the parent E14 cells. Using the EB hanging-drop method, GR-E14 cells effectively generated contracting cardiomyocytes (70%) under physiological glucose conditions. High glucose significantly delayed cardiogenesis and suppressed gene expression pertinent to cardiac lineage specification. Our study provides evidence that glucose is a crucial effector in ES cardiogenesis.

## Methods

### mESCs maintenance

The E14 mESC line and the D3 cell line derived from blastocysts [27] were purchased from ATCC (Manassas, VA, USA) and maintained in feeder-free conditions according to the ATCC instructions. Briefly, E14 mESCs were plated on 6-well culture plates coated with 0.1% gelatin in DMEM medium (DMEM, 15% ES quality FBS, 1% NEAA, 1% glutamine, 1% β-mercaptolethanol, 1000 U/ml LIF, 1% antibiotics). Medium was changed daily. Regular subculture was performed by a ratio of 1 to 6. E14 mECS line maintained under 25 mM glucose medium was initially transferred to a medium with 11.1 mM glucose for eight passages and subsequently 8.3 mM glucose for another eight passages. The resulting E14 cells were then switched into medium with the physiological glucose concentration (5 mM) for four passages. The same protocol was applied to the D3 mESC line. All of the experiments in this study began with E14 mES cells (called GR-E14) from passage 20 under 5 mM glucose conditions.

### E14 cell differentiation to cardiomyocytes

Cardiomyocytes were differentiated from GR-E14 cells via the hanging-drop method [28] in differentiation medium containing 5 or 25 mM glucose (25% DMEM, 25% F12 medium, 50% neurobasal medium, 1% N2, 2% B27, 1% NEAA, 1% glutamine, 1% β-mercaptolethanol, 1% antibiotics). GR-E14 cells at 80% confluence were dissociated with Accutase solution (Life Technologies, Grand Island, NY, USA) at 37 °C for 7 minutes, and the collected cells were adjusted to a concentration of 3.3 × 10^4^ cells/ml. Fifty drops of 30 μl cell suspension (1000 cells/drop) were seeded into the lid of a 10-cm petri dish with 15 ml PBS for EB formation for 3 days followed by suspension culture in a petri dish for an additional 2 days in medium containing 5 mM or 25 mM glucose. After 5 days, the formed EBs were transferred into 0.1% gelatin-coated 6-well culture plates and cultured for an additional 7 to 9 days for cardiogenesis. Contracting EBs and cell colonies were monitored daily under microscopy. For counting the contracting EB number, 50 EBs per group were plated on a 10-cm culture dish coated with 0.1% gelatin and were monitored daily under microscopy.

### Fluorescence-activated cell sorting (FACS)

Cells at differentiation day 6, 8, and 10 were dissociated into single cells by Trypsin-EDTA and transferred to flow cytometry tubes (BD Biosciences, San Jose, CA, USA) followed by fixation and permeabilization using the BD Cytofix/Cytoperm Kit (BD Biosciences, San Jose, CA, USA) with addition of 1:200 mouse anti-Tnnt2 antibody (Sigma-Aldrich, St. Louis, MO, USA) for 30 minutes at room temperature. Secondary antibody staining was performed in 1:1000 Alexa Fluor 488 goat anti-mouse IgG1 for 30 minutes. Cells were analyzed using the FACSCanto II and the FACSDiva software (BD Biosciences, San Jose, CA, USA). Data were analyzed using the FlowJo 10.1 software (FlowJo, LLC, Ashland, OR, USA).

### RNA isolation and real-time PCR

RNA was isolated with Trizol reagent (Invitrogen, Grand Island, NY, USA) from cells at different differentiation days according to the manufacturer’s instruction. cDNAs were synthesized from 1 μg total RNA by the Quantitect Reverse Transcription Kit (Qiagen, Valencia, CA, USA). The real-time qPCR for Oct4, Sox2, Lin28, Klf2, T, Glut2, Mixl1, Gata4, Tbx5, Nkx2.5, Mef2c, TnnT2, SERCA2A, RYR2, Hcn1-4, and Kcn1 were performed by the Rt^2^ Sybr Green Rox qPCR Mastermix (Qiagen, Valencia, CA, USA) in the StepOnePlus system (Applied Biosystems, Grand Island, NY, USA). Primer sequences for qPCR are listed in the Additional file 1: Table S1.Additional file 2: Video 1. Cardiomyocyte contraction frequency in low glucose (AVI 505 kb)
Additional file 3: Video 2. Cardiomyocyte contraction frequency in high glucose (AVI 829 kb)


### Western blotting

A total of 30–50 μg protein from cells at differentiation day 9 was extracted using the cell lysis buffer (Cat# 9803, Cell Signaling, Danvers, MA, USA) containing a 1% protease inhibitor cocktail (Sigma-Aldrich, St. Louis, MO, USA), 1% PMSF, 2% sodium fluoride, and 2% sodium orthovanadate. Immunobilon-P (EMD Milllipore, Billerica, MA, USA) membranes were used for immunoblotting. Membranes were sequentially exposed to mouse anti-GATA4 (1:1000, Origene, Rockville, MD, USA), rabbit anti-TBX5 (1:1000, Life Technologies, Grand Island, NY, USA), mouse anti-MEF2C (1:1000, Thermo Fisher Scientific, Waltham, MA, USA), rabbit anti-KCN1 (1:1000, Prosci Inc., San Diego, CA, USA) and rabbit anti-TnnT2 (1:1000, Origene, Rockville, MD, USA) overnight followed by incubation of goat anti-rabbit IgG or goat anti-mouse IgG conjugated with HRP (1:10,000, Jackson ImmunoResearch Laboratories, West Grove, PA, USA) for 4 hours. Signals were detected using the SuperSignal West Femto Maximum Sensitivity Substrate Kit (Pierce Biotechnology, Rockford, IL, USA) and chemiluminescence emitted from the bands was directly captured using the UVP Bioimage EC3 system. Densitometric analyses of chemiluminescence signals were performed using the VisionWorks LS software (UVP, Upland, CA, USA).

### Immunofluorescence

EBs were plated onto 0.1% gelatin-coated chamber slides (Thermo Fisher Scientific, Waltham, MA, USA) in high or low glucose medium. At differentiated day 7 to 9, cells were fixed by 4% paraformaldehyde for 10 minutes at room temperature followed by permeabilization with 0.05% TritonX-100 for 10 minutes. Samples were blocked for 1 hour with 10% heat-inactivated donkey serum in PBS and incubated with the following antibodies: TNNT2 (1:200, Origene, Rockville, MD, USA), NKX2.5 (1:200, Sigma-Aldrich, St. Louis, MO, USA), and HCN1 (1:200, Sigma-Aldrich, St. Louis, MO, USA), overnight at 4 °C. After washing with PBS three times, cells were incubated with a Cy3-conjugated secondary antibody (diluted 1:1000) at room temperature for 4 hours followed by incubation with DAPI for 10 minutes and mounted with aqueous mounting medium. Fluorescence image were taken on Nikon H600L microscope (Nikon, Tokyo, Japan) with the IPLab imaging system (Scientific Instrument Company, San Diego, CA, USA).

### Ca^2+^ imaging

Differentiated EB in low or high glucose conditions were placed on gelatin-coated chamber slides (Lab-Tek, Thermo Fisher Scientific, Waltham, MA, USA) for Ca^2+^ imaging based on a previously described protocol [29]. ES-derived cardiomyocytes at day 9 were loaded with 5 μM fluo 4-AM (Thermo Fisher Scientific, Waltham, MA, USA) for 20 minutes at room temperature and replaced with normal Tyrode’s solution containing (in mM) 140 NaCl, 10 Hepes, 0.5 MgCl_2_, 0.33 NaHPO_4_, 5.5 glucose, 1.8 CaCl_2_, and 5 KCl (pH 7.4). Confocal imaging (Zeiss LSM-510; Carl Zeiss, Oberkochen, Germany) was used to monitor Ca^2+^ signals using a line scan method for 10,000 time at 1.93 msec per line. Data were analyzed using ImageJ (NIH, Bethesda, MD, USA).

### Statistical analysis

Data was analyzed by the Sigmaplot 12.5 software (Systat Software Inc, San Jose, CA, USA). Experiments were repeated independently three times. Data are presented as mean ± SD. Statistical differences were evaluated using one-way analysis of variance (ANOVA) followed by the Tukey test.

## Results

### E14 cells adapted to physiological glucose concentrations retain stemness and have a high expression of glucose transporter 2

**Fig. 1:**
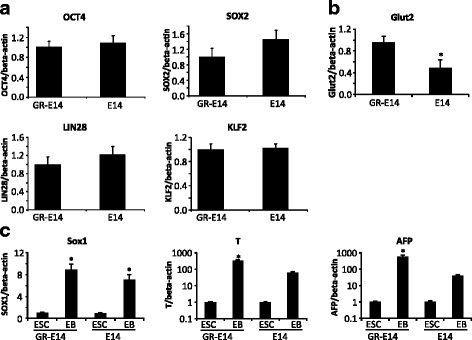
Characteristics of parent E14 cells and the GR-E14 cells adapted to low glucose. **a** mRNA levels of pluripotent markers, OCT4, SOX2, KLF2, and LIN28, in parent E14 cells and E14 cells adapted to low glucose (GR-E14 cells). **b** Glut2 mRNA levels in GR-E14 and parent E14 cells. **c** mRNA levels of ectoderm marker SOX1, mesoderm marker T, and endoderm marker AFP in EBs. *E14* E14 cells maintained under 25 mM glucose condition, *EB* embryoid body derived from E14 or GR-E14 cells, *GR-E14* glucose-responsive E14 cell line, which was gradually adapted to 5 mM glucose medium from 25 mM glucose medium. All experiments were repeated three times (n = 3), the value was dedicated as mean ± SD. ^*^Indicates significant difference compared with the other group(s)

Like most ES cell lines, the E14 cell is maintained under high glucose conditions (25 mM glucose). In order to assess the effect of high glucose on ES cell differentiation, it is essential to establish an ES cell line which is adapted to the physiological glucose concentration (5 mM). Here, we showed a simple and cost-saving method for the establishment of an E14 cell line adapted to physiological glucose conditions by gradually lowering the glucose concentration to 5 mM in culture medium as described in the “Methods” section. The expression of pluripotent markers, OCT4, SOX2, LIN28, and KLF2, in the glucose-adapted E14 cell line was similar to that in the parent E14 cell line that was maintained under high glucose conditions (Fig. 1a). It has been shown that an ES cell line established under low glucose conditions expresses higher levels of glucose transporter 2 (Glut2) comparing with those in an ES cell line cultured in high glucose [30]. Glut2 expression was significantly higher in the low glucose-adapted E14 cells than in the parent E14 cells (Fig. 1b), suggesting that the low glucose-adapted E14 cells are glucose responsive (GR-E14 cells). Embryoid bodies derived from GR-E14 cells had a similar expression of SOX1, Brachyury (T), and AFP as that in the parent E14 cells (Fig. 1c), indicating that the GR-E14 cells retained the capability of differentiation into all three primary germ layers.

### High glucose suppresses the differentiation of GR-E14 cells into contracting cardiomyocytes

**Fig. 2:**
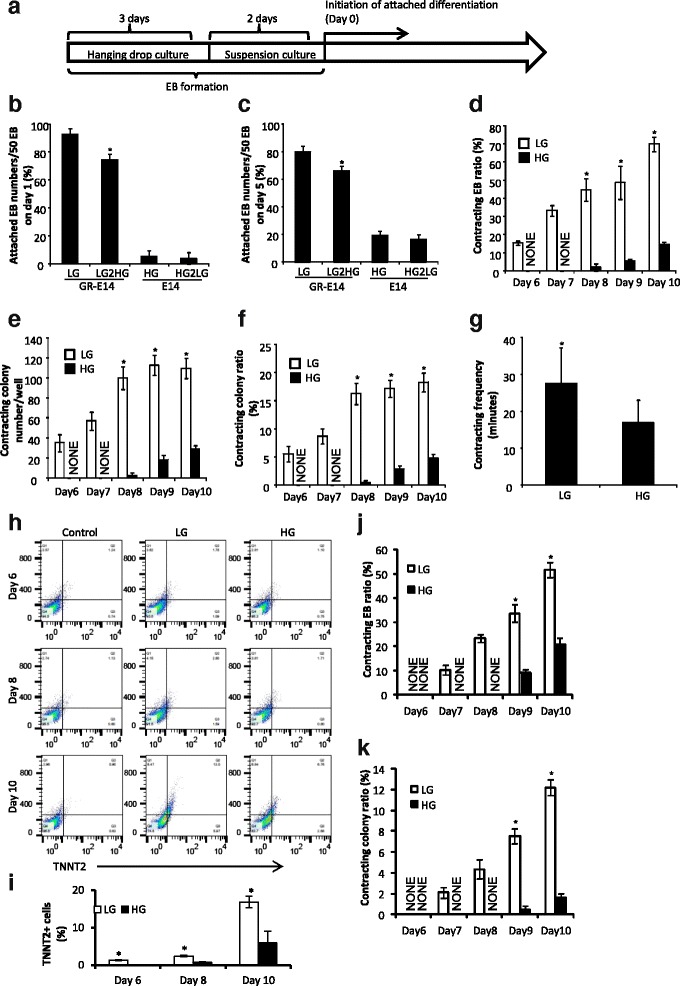
High glucose suppresses GR-E14 cell cardiogenesis. **a** Schematic diagram of ES cell cardiomyocytes. Hanging-drop culture was performed with one drop of 30 μl medium per 1000 cells for 3 days for EB formation, and then suspension culture was done in a 10-cm petri dish for another 2 days for EB growth followed by differentiation. **b** The numbers of EB attached to the culture plate on day 1 after seeding the formed EBs (differentiation). **c** The numbers of EB attached to the culture plate on day 5 of differentiation. GR-E14 cells adapted to low glucose were used for hanging-drop culture in low glucose (5 mM, LG) or high glucose (25 mM) medium (*LG2HG*), parent E14 cells maintained in high glucose medium were used for hanging-drop culture in HG or low glucose medium (*HG2LG*). **d** The percentage of contracting EBs in GR-E14 cells under LG/HG condition. Fifty EBs per group (n = 3) were used for cardiogenesis and the contracting EB percentage was calculated by the formula: (number of contracting EBs/number of total EBs) × 100%. **e** Numbers of contracting colonies from GR-E14 cells under LG/HG conditions. At day 5 of EB formation, an aliquot of 10 μl EB suspension, containing 20 EBs (equivalent to 5 × 10^5^ cells), was seeded into a well of a 6-well culture plate for cardiogenesis. **f** Percentage of contracting colonies from GR-E14 cells under LG/HG conditions. The contracting colony percentage was calculated by the formula: (number of contracting colonies/number of total colonies) × 100%. **g** Frequencies of contracting colonies from GR-E14 under LG/HG conditions. **h** The frequencies of TNNT2^+^ cardiomyocytes at day 6 (*left*), day 8 (*middle*) and day 10 (*right*) after differentiation. *HG* high glucose (25 mM, *red*), *LG* low glucose (5 mM, *gray*),. **i** Percentages of TNNT2^+^ cardiomyocytes differentiated from GR-E14 cells. TNNT2^+^ cell percentages (%) = (TNNT2^+^ cell number/total cell number) × 100%. Total cell number is 10,000 per group. **j** The percentage of contracting EBs in D3 cells under LG/HG conditions. The protocol was the same as that of GR-E14. **k** Percentage of contracting colonies from D3 cells under LG/HG conditions. The protocol was the same as that of GR-E14. *E14* E14 cells maintained under 25 mM glucose condition, *GR-E14* glucose-responsive E14 cell line, which was gradually adapted to 5 mM glucose medium from 25 mM glucose medium. All experiments were repeated three times (n = 3). Data were expressed as mean ± SD. ^*^Indicate significant difference compared with the other group(s)

CHDs frequently occur in babies whose mothers have diabetes [31]. Previous studies have demonstrated that the high glucose of diabetes suppresses gene expression related to apoptosis, proliferation, and migration in the developing heart [13, 31, 32]. However, early development events such as the ontogeny of cardiomyocytes from ES cells may be affected by high glucose and thus contribute to the etiology of CHD formation in diabetic pregnancies. We hypothesize that high glucose suppresses ES cardiogenesis. To test this hypothesis, embryoid bodies (EBs) were formed for 5 days with the hanging-drop method prior to further differentiation into cardiomyocytes [33, 34], (Fig. 2a). When the parent E14 cells, which are accustomed to high glucose, were used for differentiation into cardiomyocytes under either low glucose (5 mM) or high glucose (25 mM) conditions, very few EBs derived from these cells could attach to surfaces of culture plates coated with 0.1% gelatin at the first day of differentiation (Fig. 2b). Even after 5 days, only few EBs attached to culture plates (Fig. 2c), and none of these attached EBs could differentiate to contracting cardiomyocytes (data not included). In contrast, most of the EBs derived from GR-E14 cells (93.3 ± 4.6% in 5 mM glucose and 74.0 ± 4.0% in 25 mM glucose) attached to culture plates at the first day of differentiation and remained attached at high numbers in day 5 (Fig. 2b and c). Therefore, we only focused on GR-E14 for further experiments to assess the effect of high glucose.

For the differentiation of GR-E14 cells, contracting EBs or cardiomyocytes appeared at day 6 in 5 mM glucose and at day 8 in high glucose (25 mM) medium, respectively (Fig. 2d). Thus, high glucose delayed GR-E14 cell cardiogenesis for 2 days (Fig. 2d). Whereas 70.0 ± 4.0% of the EBs were differentiated into contracting cardiomyocytes in 5 mM glucose at day 10, only 14.7 ± 1.2% contracting EBs were obtained under high glucose (25 mM) conditions (Fig. 2d).

The contracting colony number in 5 mM glucose at day 8 of differentiation was significantly higher than that in high glucose (Fig. 2e). At day 10 of differentiation, the contracting colony number in 5 mM glucose (109.7 ± 10.2) was significantly higher than that under high glucose condition (29.0 ± 4.0), (*P* < 0.01) (Fig. 2e). Moreover, the percentage of contracting colonies in 5 mM glucose achieved 5.53 ± 1.35% at day 6. However, it took 10 days to achieve a similar contracting colonies ratio (4.83 ± 0.67%) in 25 mM glucose (Fig. 2f). Furthermore, the contracting frequency of contracting colonies in 5 mM glucose was significantly higher than that in 25 mM glucose at day 10 of differentiation (Fig. 2g and Additional file 2: Video 1 and Additional file 3: Video 2).

To further determine if cardiogenesis was affected by high glucose, FACS was performed to detect TNNT2 frequencies in differentiated cells. Consistent with our finding above, TNNT2-positive cells emerged at day 6 in 5 mM glucose, but were not present in 25 mM glucose (Fig. 2h and i). Additionally, the TNNT2-positive cell number in 25 mM glucose was significantly lower than that in 5 mM glucose medium at day 8. 16.8 ± 1.5% of TNNT2-positive cardiomyocytes were observed in 5 mM glucose at day 10, whereas only 6.0 ± 3.1% of TNNT2-positive cardiomyocytes were obtained in 25 mM glucose medium. Collectively, these data demonstrate that high glucose suppresses GR-E14 cell cardiogenesis.

To further confirm our finding, another mouse ES cell line, D3, was adapted into low glucose and differentiated into cardiomyocytes using the same method described above. For the differentiation of D3 cells, contracting EBs or cardiomyocytes appeared at day 7 in 5 mM glucose and at day 9 in high glucose (25 mM) medium, respectively (Fig. 2j, k). Thus, consistent with the observation in GR-E14 cells (2d, e), high glucose delayed D3 cell cardiogenesis for 2 days (Fig. 2d). 51.7 ± 3.1% of the EBs were differentiated into contracting cardiomyocytes in 5 mM glucose at day 10, whereas only 21.0 ± 1.6% contracting EBs were obtained under high glucose (25 mM) conditions (Fig. 2j). Furthermore, the contracting colonies ratio in 25 mM glucose was also significantly lower than that in 5 mM glucose (Fig. 2k). However, the differentiation efficiency of the D3 cell line was lower than that of GR-E14 (Fig. 2d, j), the following experiments were performed on GR-E14 cells only.

### High glucose represses gene expression essential for cardiogenesis

**Fig. 3:**
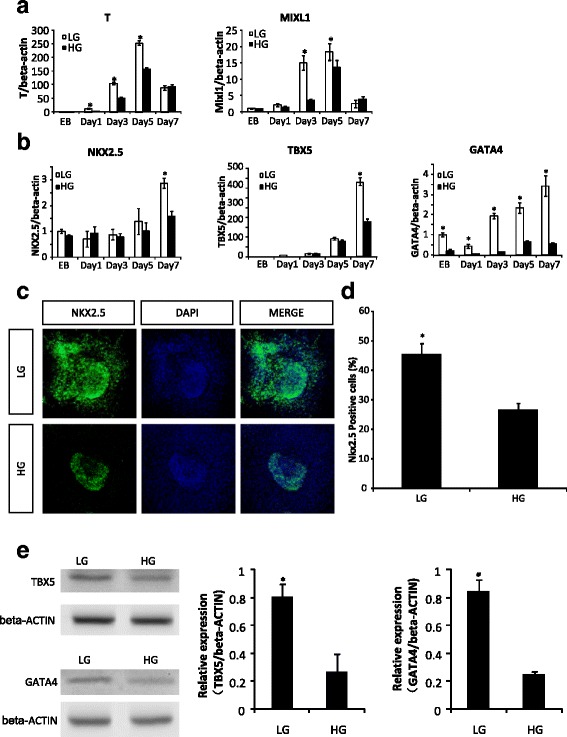
High glucose inhibits gene expression essential for cardiogenesis. **a** mRNA levels of mesodermal markers, T, and MIXL1 during GR-E14 cell cardiogensis. **b** mRNA levels of cardiac transcription factors, NKX2.5, TBX5, and GATA4. **c** Representative images of NKX2.5 immunofluorescent staining (*green*). DAPI (*blue*) was for cell nuclei staining. **d** Quantitative fluorescent density data of NKX2.5 immunofluorescent staining. **e** TBX5 and GATA4 protein levels at differentiation day 7. Gene expression was assessed at EB formation day 5 and attachment culture (differentiation) at day 1, 3, 5, and 7. Experiments were repeated three times (n = 3). *EB* embryoid body derived from E14 or GR-E14 cells, *HG* high glucose (25 mM), *LG* low glucose (5 mM). Values were dedicated as mean ± SD. ^*^Indicates significant difference compared with the other group

To determine whether high glucose affects the expression of early cardiac markers, we detected precardiac mesoderm marker, Brachyury (T) and Mixl1, and heart field-specific progenitor markers, NKX2.5, TBX5, and GATA4. The expression of T and Mixl1 in 5 mM glucose was significantly higher than that in high glucose (25 mM) before day 5 of differentiation (Fig. 3a). More interestingly, the expression of heart field-specific progenitor markers, NKX2.5, TBX5, and GATA4, in 5 mM glucose was significantly higher than that in high glucose (Fig. 3b). Specifically, GATA4 expression in 5 mM glucose was significantly higher than that in high glucose at the EB stage and throughout the entire course of differentiation (Fig. 3b). Furthermore, 45.4 ± 3.7% of NKX2.5-positive cells were observed in 5 mM glucose; however, 26.4 ± 2.3% cells were NKX2.5-positive in high glucose (Fig. 3c, d). TBX5 and GATA4 protein levels were also suppressed by high glucose (Fig. 3e). Thus, high glucose suppresses cardiac gene expression during GR-E14 cardiogenesis.

### High glucose inhibits the maturation of differentiated cardiomyocytes

**Fig. 4:**
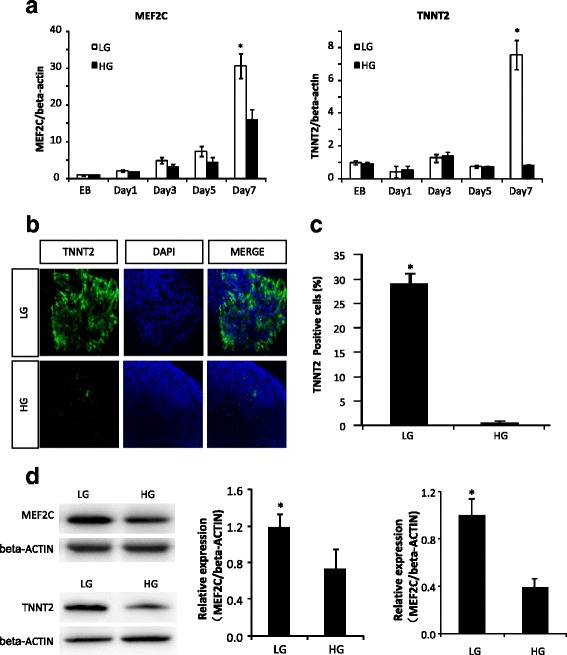
High glucose suppresses cardiomyocyte-specific marker expression. **a** mRNA levels of cardiomyocyte structure proteins, MEF2C, and TNNT2. **b** Representative images of TNNT2 immunofluorescent staining. **c** Quantitative fluorescent density data of TNNT2 immunofluorescent staining. **d** MEF2C and TNNT2 protein expression at differentiation day 9. Gene expression was assessed at EB formation day 5 and differentiation day 1, 3, 5, and 7. Experiments were repeated three times (n = 3). *EB* embryoid body derived from E14 or GR-E14 cells, *HG* high glucose (25 mM), *LG* low glucose (5 mM). Values were dedicated as mean ± SD. ^*^Indicates significant difference compared with the other group

Because high glucose significantly decreased the number of contracting cardiomyocytes, we next assessed the expression of mature cardiomyocyte markers, TNNT2 and MEF2C, during differentiation of GR-E14 cells into cardiomyocytes. At day 7 of differentiation, MEF2C and TNNT2 levels were significantly higher in 5 mM glucose than those in high glucose (Fig. 4a). Coinciding with mRNA expression, TNNT2 immunofluorescent staining showed a significantly higher number of TNNT2-positive cells in 5 mM glucose compared with high glucose (Fig. 4b, c). Total cellular MEF2C and TNNT2 protein levels were significantly reduced by high glucose (Fig. 4d).

These findings demonstrate that cardiomyocyte maturation is attenuated by high glucose.

### High glucose reduces the expression of potassium channel proteins that are essential for cardiomyocyte contraction

**Fig. 5:**
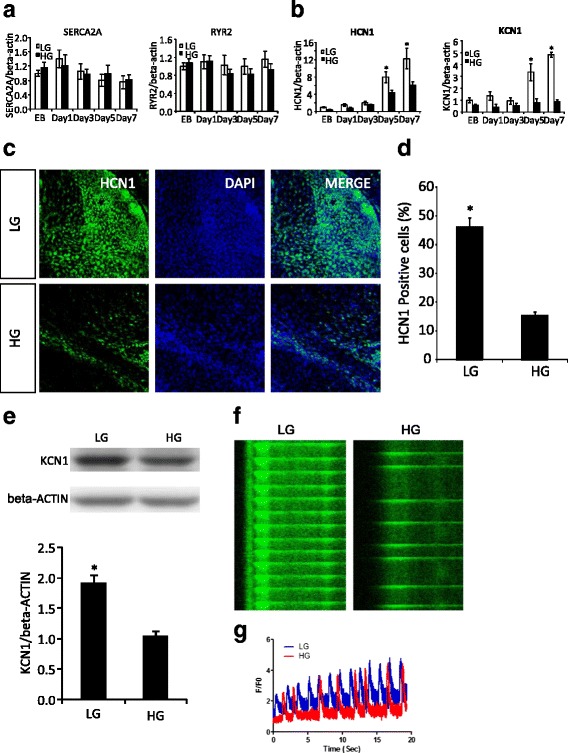
The inhibitory effect of high glucose on calcium-handling and potassium channel gene expression during GR-E14 cardiogenesis. **a** mRNA levels of calcium-handling genes, SERCA2A and RYR2. **b** mRNA levels of potassium channel genes, HCN1 and KCN1. **c** Representative images of HCN1 immunofluorescent staining (*green*). DAPI (*blue*) was for cell nuclei staining. **d** Quantitative fluorescent density data of HCN1 immunofluorescent staining. **e** KCN1 protein expression at differentiation day 9. Gene expression was assessed at EB formation day 5 and differentiation day 1, 3, 5, and 7. **f** Line scan image of fluo 4 in ES cell-derived cardiomyocytes in low or high glucose conditions. **g** Ca^2+^ wave frequency of ES-derived cardiomyocytes in low or high glucose conditions. Experiments were repeated three times (n = 3). *EB* embryoid body derived from E14 or GR-E14 cells, *HG* high glucose (25 mM), *LG* low glucose (5 mM). Values were dedicated as mean ± SD. ^*^Indicates significant difference compared with the other group

Cardiomyocyte contracting function is highly associated with calcium-handling process and several ion channels [35, 36]. Among them, the hyperpolarization-activated cyclic nucleotide-modulated (HCN) channels contribute to a faster rhythmicity of beating mouse cardiomyocytes derived from ES cells [37, 38]. To understand the impaired contraction of cardiomyocytes by high glucose, we detected the expression of several ion channels genes, including Cav1.2, Nav1.5, HCN1-4, KCN1, and calcium-handling genes, SERCA2A and RYR2. High glucose did not affect the expression of the two main calcium-handling genes, SERCA2A and RYR2 (Fig. 5a). However, the expression of the potassium channels, HCN1 and KCN1, was significantly decreased by high glucose beginning at day 5 of differentiation before ontogeny of beating cardiomyocyte at day 6 (Fig. 5b). Moreover, there are more HCN1-positive cells in 5 mM than in high glucose (Fig. 5c and d). Total cellular KCN1 protein levels were significantly reduced by high glucose (Fig. 5e). Furthermore, the functionality of ES-derived cardiomyocytes under high glucose condition was determined by Ca^2+^ wave profile. High glucose significantly suppressed the frequency of Ca^2+^ wave in differentiated cardiomyocytes (Fig. 5f and g). Consistent with the reduced Ca^2+^ wave frequency, high glucose inhibited the contracting frequency of contracting colonies (Fig. 2g and Additional file 2: Video 1, and Additional file 3: Video 2). These results indicate that the decreased potassium channel expression may contribute to the impaired contraction of cardiomyocytes in high glucose.

## Discussion

The precise temporal regulation of thousands of genes that govern developmental decisions is required during heart development [39]. However, gene expression patterns during developmental transitions to cardiac lineage are still not well understood. Cardiomyocyte differentiation derived from ES cells is a widely used model for studies in mimicking early cardiomyocyte development in vivo. There is a number of robust protocols for ES cell cardiogenesis including the EB hanging-drop method, which have provided many pivotal clues for understanding heart lineage specification [25]. Surprisingly, most ES cell maintenance and differentiation conditions are at high glucose conditions, often with 25 mM glucose [40]. High glucose may adversely impact ES cardiogenesis. The effect of glucose on ES cell cardiogenesis has been previously assessed [40]. It has been shown that 25 mM glucose supported ES cardiogenesis through reactive oxygen species (ROS), whereas 5 mM glucose did not generate any contracting cardiomyocytes [40]. However, the ES cells in this previous study were generated and maintained under high glucose and just adapted to low glucose for a short time, two passages, prior to differentiation [40]. Due to this short low glucose adaptation, the ES cells may not acquire glucose responsiveness. Indeed, they did not assess glucose responsiveness of ES cells before differentiation [40]. Therefore, whether these short-term low glucose-adapted cells responded to high glucose was unknown. In contrast, we obtained an ES cell line, GR-E14, which is adapted to low glucose condition through a 20 passage graduated glucose reduction, and acquires glucose responsiveness before differentiation. The GR-E14 cells can propagate in a long-term fashion without losing any stemness and pluripotency. A recent report demonstrated that ES cells established under low glucose condition possessed glucose responsiveness similar to cells of preimplantation and early postimplantation embryos by manifesting high Glut2 expression [30]. The GR-E14 cell line also expresses high levels of Glut2, implicating its glucose responsiveness. Indeed, subsequent studies demonstrated that high glucose adversely affected GR-E14 cardiogenesis.

The parent E14 cells accustomed to high glucose failed to generate any contracting cardiomyocytes using the hanging-drop method. It has been reported that ES cells generated and maintained under high glucose conditions can be differentiated into contracting cardiomyocytes [28]. ES cell cardiogenesis depends on the differentiation microenvironment [41], especially the starting ES cell number, the volume of hanging drops as well as the time of EB adherence to gelatin-coated plates [42]. The discrepancy between our study and those of others may be due to different settings of the hanging-drop method. In Crespo’s study, ES cells were only passaged twice in low (5 mM) glucose medium [40]. However, in the present study, ES cells were passaged 20 times with gradually decreased glucose levels. GR-E14 cells were completely adapted in low glucose conditions and had a high pluripotent potential. Second, the EB formation protocols between ours and Crespo’s were different. They performed 2 days of EB formation in hanging drop followed by adhesion differentiation on gelatin-coated dishes. However, 5 days of EB formation protocol including 3 days hanging drop and 2 days suspension culture were performed in our study. Third, the hanging-drop settings were also different. Settings of 1000 cells/30 μl/drop were used in the present study for 3 days, whereas 1000 cells/20 μl/drop were used in Crespo’s study for 2 days. All of these differences may account for the discrepancy between the two studies. Nevertheless, GR-E14 cells can be differentiated into contracting cardiomyocyte at very high efficiency and GR-E14 cell cardiogenesis is subjected to glucose regulation, suggesting that our hanging-drop method settings are ideal for studies in glucose regulation of ES cell cardiogenesis. High glucose suppresses GR-E14 cell cardiogenesis by delaying the ontogeny of contracting EBs and reducing EB contraction frequencies. Previous studies have shown that heart rates are lower in the offspring of diabetic mothers than nondiabetic mothers [43]. Thus, the GR-E14 cell line is a useful in vitro model in mimicking the abnormal cardiogenesis of embryos exposed to pregestational diabetes.

CHDs are the most common defects in offspring of diabetic mothers [31]. Previous studies have shown that gene dysregulation is critically involved in diabetes-induced CHDs [13]. Our previous studies have determined that oxidative stress, endoplasmic reticulum stress and pro-apoptotic kinase signaling mediate the teratogenicity of diabetes in the developing heart by modulating gene expression [4–6, 8]. To reveal the etiology of diabetes-induced CHDs, it is critical to understand the gene alterations during early cardiogenesis. A highly conserved gene regulatory network controls the initial differentiation, proliferation, and maturation of cardiomyocytes [21]. The anteriorly migrated mesoderm cells, once received appropriate signals, switch on a highly conserved cardiac transcriptional program via sequentially expressing heart-specific transcription factors [44]. Initially, Brachyury (T) and Mixl1-positive mesodermal precursor cells enter a precardiac mesoderm stage as evidence of Mesp1 expression [45, 46]. Subsequently, Mesp1-positive cells begin to express NKX2.5 and TBX5, which combined with GATA4 to activate cardiac structural genes such as TnnT2, MHC, and MLC [44]. Any abnormalities in this cardiac specification program result in CHDs. Mesodermal precursor cell markers, T and Mixl1, were suppressed by high glucose during early GR-E14 cardiogenesis. T deficiency in mice resulted in early embryonic lethality due to defects in mesoderm formation [47]. Mixl1 is required for axial mesendoderm morphogenesis in differentiating ES cells and murine embryos [48, 49]. Suppression of both T and Mixl1 may be a primary cause of abnormality in GR-E14 cardiogenesis by high glucose. Nkx2.5, the earliest known marker of the cardiac lineage in vertebrate embryos, is expressed in mouse embryo from E7.5 onward and its deletion causes CHDs resembling those in diabetic pregnancies [50, 51]. NKX2.5, TBX5, and GATA4 expression were suppressed by high glucose. Consistent with the downregulation of these three cardiac transcription factors, high glucose suppressed the expression of mature cardiomyocyte markers, TNNT2 and MEF2C. In human, mutation of TnnT2 is tightly associated with hypertrophic cardiomyopathy, dilated cardiomyopathy, and left ventricular noncompaction cardiomyopathy [52–54]. Suppression of TnnT2 may contribute to impaired GR-E14 cardiogenesis under high glucose condition. These findings support the hypothesis that high glucose adversely impacts the entire ES cardiogenesis program. Our recent studies have demonstrated that cellular stress including oxidative stress and endoplasmic reticulum (ER) stress mediates the adverse effect of high glucose in the developing heart [8, 9]. Future studies will aim to determine whether cellular stress mediates the inhibitory effect of high glucose in ES cell cardiogenesis.

Intracellular Ca^2+^ is the central regulator of cardiac contractility [55]. It is critically important how Ca^2+^ is regulated during initiation of contraction of cardiomyocyte under high glucose condition. In the present study, some key regulation proteins in cardiac contractility, including calcium, sodium, potassium channel proteins, and calcium in/outflux proteins (RYR2 and SERCA2A), were investigated; however, most of these genes expressed at similar levels under low and high glucose conditions. Two potassium channel proteins, HCN1 and KCN1, were suppressed by high glucose. In particularly KCN1 was almost completely suppressed by high glucose. The HCN1 protein is highly expressed in the sinoatrial node and is colocalized with HCN4, the main sinoatrial pacemaker channel isoform [56]. Therefore, high glucose-suppressed HCN1 and KCN1 may contribute to reduced number of contracting cardiomyocytes derived from GR-E14 cells. It is well known that intracellular calcium release from the sarcoplasmic reticulum is required for heart contraction [57]. Since high glucose inhibited ES cell-derived cardiomyocytes contraction, it is important to investigate the relationship between Ca^2+^ wave profile and contraction frequency. Interestingly, reduced frequency of Ca^2+^ wave profile under high glucose condition was consistent with slower contraction frequency in differentiated cardiomyocytes compared to that in low glucose condition. These findings suggest that the functionality of cardiomyocytes derived from ES cells is impaired by high glucose.

Our finding indicated that high glucose inhibited GR-E14 cell differentiation into cardiomyocytes. However the underlying mechanism needs to be further elucidated. Jang et al*.* reported that high glucose upregulated ES cell pluripotency through elevated level of O-linked-N-acetylglucosamine in proteins of core components of the pluripotency network [58]. Furthermore, ES cells utilize glucose catabolism to maintain a high level of intracellular α-ketoglutarate, promoting histone/DNA demethylation and pluripotency [59]. Therefore suppression of ES differentiation by high glucose may be due to high glucose-increased pluripotency, which may ultimately delay ES differentiation. Only one study indicates that high glucose-induced reactive oxygen species (ROS) promotes cardiomyocyte differentiation from ESC cells [40]. Other studies have shown that high glucose enhances the expression of pluripotent markers, OCT4, NANOG, and SOX2, in adipose-derived stem cells through ROS generation [60, 61]. This indirect evidence supports our conclusion that high glucose impairs ES cell differentiation into cardiomyocytes.

## Conclusions

GR-E14 cells adapted to physiological glucose acquire glucose responsiveness. The GR-E14 cell line may be a useful model for investigating the effect of high glucose on early cardiac specification. Our study showed for the first time that high glucose delayed and impaired GR-E14 cardiomyocytes by inhibiting early cardiac specification genes, cardiac transcription factors, and genes essential for cardiomyocyte maturation and contractility. Thus, high glucose inhibits ES cardiogenesis by suppressing gene expression. Our findings guide future ES maintenance and differentiation by showing the adversity of high glucose in ES cardiogenesis and providing the beneficial effects using physiological concentration of glucose at 5 mM.

## Additional files

Additional file 1: Table S1. Primer sequences for RT-qPCR. (DOCX 13 kb)
