# Supplementary material for: High glucose suppresses embryonic stem cell differentiation into cardiomyocytes: High glucose inhibits ES cell cardiogenesis
Source: Stem Cell Res Ther. 2016 Dec 9;7:187. doi: 10.1186/s13287-016-0446-5 (PMC5148851; doi:10.1186/s13287-016-0446-5)
Supplement: Additional file 1: Table S1. — Primer sequences for RT-qPCR. (DOCX 13 kb) [file 13287_2016_446_MOESM1_ESM.docx]

**High glucose suppresses embryonic stem cell differentiation into cardiomyocytes**

Penghua Yang^1^, Sunjay Kaushal^2^, Peixin Yang^1, 3^

***Author Affiliations:***

^1^Department of Obstetrics, Gynecology & Reproductive Sciences, ^2^Division of Cardiac Surgery, ^3^Department of Biochemistry & Molecular Biology, University of Maryland School of Medicine

Baltimore, MD 21201, USA.

| **Supplementary Table 1. Primer sequences for RT-qPCR** | | |
| --- | --- | --- |
| Gene | Forward primer | Reverse primer |
| OCT4 | TTGGGCTAGAGAAGGATGTGGTT | GGAAAAGGGACTGAGTAGAGTGTGG |
| SOX2 | GCACATGAACGGCTGGAGCAACG | TGCTGCGAGTAGGACATGCTGTAGG |
| LIN28 | GGCATCTGTAAGTGGTTCAACG | CCCTCCTTGAGGCTTCGGA |
| KLF2 | CTCAGCGAGCCTATCTTGCC | CACGTTGTTTAGGTCCTCATCC |
| SOX1 | AAGGAACACCCGGATTACAAGT | GTTAGCCCAGCCGTTGACAT |
| T | GCTTCAAGGAGCTAACTAACGAG | CCAGCAAGAAAGAGTACATGGC |
| AFP | CTTCCCTCATCCTCCTGCTAC | ACAAACTGGGTAAAGGTGATGG |
| GLUT2 | TCAGAAGACAAGATCACCGGA | GCTGGTGTGACTGTAAGTGGG |
| MIXL1 | ACGCAGTGCTTTCCAAACC | CCCGCAAGTGGATGTCTGG |
| NKX2.5 | GACAAAGCCGAGACGGATGG | CTGTCGCTTGCACTTGTAGC |
| TBX5 | ATGGCCGATACAGATGAGGG | TTCGTGGAACTTCAGCCACAG |
| GATA4 | CCCTACCCAGCCTACATGG | ACATATCGAGATTGGGGTGTCT |
| MEF2C | GTCAGTTGGGAGCTTGCACTA | CGGTCTCTAGGAGGAGAAACA |
| TNNT2 | GAGCTACAGACTCTGATCGAGG | CCGCTCATTGCGAATACGC |
| RYR2 | ACGGCGACCATCCACAAAG | AAAGTCTGTTGCCAAATCCTTCT |
| SERCA2A | TGGAACAACCCGGTAAAGAGT | CACCAGGGGCATAATGAGCAG |
| HCN1 | CAAATTCTCCCTCCGCATGTT | TGAAGAACGTGATTCCAACTGG |
| KCN1 | ATGAGCCTGCCCAATTCCAC | GAGCTGAGACTTACGAGCCA |

Video 1. Cardiomyocyte contraction frequency in low glucose

Video 2. Cardiomyocyte contraction frequency in high glucose
